# Supplementary material for: Natural hybridization in heliconiine butterflies: the species boundary as a continuum
Source: BMC Evol Biol. 2007 Feb 23;7:28. doi: 10.1186/1471-2148-7-28 (PMC1821009; doi:10.1186/1471-2148-7-28)
Supplement: Additional File 1 — Hybrids between species of Heliconius and Eueides butterflies: a database. HTML file linking to database of all known wild-caught interspecific hybrid specimens in the Heliconiina, consisting of introductory text, a list of specimens, together with collection data and photographs of the specimens, and links to information about some artificial hybrids and mutants in the group. This is an edited copy of our online database of Heliconius hybrids [102]. To view database, download zip file and extract to a separate folder, then open index.html within that folder. [file 1471-2148-7-28-S1.zip › hybtab.html]

Wild-caught hybrids among Heliconius and Eueides

**Wild-caught
hybrids among
*Heliconius* and *Eueides* species**
  
© James Mallet, Walter Neukirchen and Mauricio
Linares 1997-2006   
**Click on hybrid number to see pictures**  
Back to hybridization document
  
J. Mallet home page
  
**Last updated:** Wednesday 30 July 2006

|  |  |  |  |  |  |  |  |  |  |
| --- | --- | --- | --- | --- | --- | --- | --- | --- | --- |
| No | Species 1 | Species 2 | m/f | Country | Locality | Year | Collection | Author | Interpretation |
| 1 | E. lybia lybia | E. ?vibilia unifasciatus | m | Brazil | Benjamin Constant |  | MN/UFRJ |  | ?F1 |
| 2 | E. isabella eva | E. vibilia vialis | m | Mexico | Veracruz: Vigía | 1978 | UNAM |  | F1 |
| 3 | E. isabella eva | E. vibilia vialis | f | Mexico | Oaxaca: Chiltepec | 1976 | UNAM |  | F1 |
| 4 | E. isabella eva | E. vibilia vialis | m | Mexico | Oax: Sierra Juárez Metatos | 1978 | UNAM |  | F1 |
| 5 | E. isabella eva | E. vibilia vialis | m | Mexico | Oaxaca: Chiltepec | 1975 | UNAM |  | F1 |
| 6 | E. isabella eva | E. procula vulgiformis | m | Costa Rica | Cartago: Juan Viñas | 1902 | MCZ |  | F1 |
| 7 | E. pavana | E. vibilia vibilia | m | Brazil | ?Rio de Janeiro |  | MN/UFRJ |  | F1 |
| 8 | H. numata superioris | H. melpomene melpomene | m | Brazil | Rio Arapiuns | 1980 | Wien | Holzinger & Holzinger 1994: 24,5c | F1 |
| 9 | H. numata ?superioris | H. melpomene nr. meriana | m | Brazil | ?Obidos |  | MN/UFRJ |  | F1? |
| 10 | H. numata ?superioris | H. melpomene nr. meriana | m | Brazil | Pará: Obidos | 1985 | Neukirchen |  | F1? |
| 11 | H. numata aurora | H. melpomene malleti | f | Peru | Loreto: Río Itaya | 1997 | Neukirchen |  | F1? |
| 12 | H. hecale vetustus | H. m. melpomene x thelxiopeia | m | Guyane | St. Laurent | <1932 | BM | Ackery & Smiles 1976:123 | F1 |
| 13 | H. hecale fortunatus | H. m. melpomene melpomene | m | Brazil | ?Obidos |  | MN/UFRJ |  | F1 |
| 14 | H. ?hecale | H. elevatus zoelleri | f | Colombia | Guainia: Puerto Inírida | 1974 | Schmidt-M/IAvH | Velez & Salazar 1991: 132 | BC->elevatus |
| 15 | H. hecale versicolor | H. elevatus pseudocupidineus | m | Peru | Loreto: Yurimaguas | 1919 | Wien | Holzinger & Holzinger 1994: 27,1b | BC->elevatus |
| 16 | H. hecale zeus | H. elevatus perchlorus | m | Bolivia | Chaparé: Todos Santos | 1969 | Wien |  | F1 |
| 17 | H. ethilla nr. metalilis | H. melpomene melpomene | m | ?Colombia |  | <1867 | BM | Ackery & Smiles 1976:173 | F1 |
| 18 | H. ethilla nr. metalilis | H. melpomene melpomene | f | Colombia | Meta: Río Negro | 1970 | Schmidt-M/IAvH | Brown 1976: 15b | F1? |
| 19 | H. ethilla nr. metalilis | H. melpomene melpomene | m | Colombia | Meta: Cubarral | 1979 | Schmidt-M/IAvH |  | BC->ethilla |
| 20 | H. ethilla nr. metalilis | H. melpomene melpomene | f | Colombia | Meta: Cubarral | 1979 | Schmidt-M/IAvH |  | BC->ethilla |
| 21 | H. ethilla metalilis | H. numata peeblesi | m | Venezuela | Barinas: Barinitas | <1908 | BM |  | F1? |
| 22 | H. ethilla narcaea | H. numata ethra | f | Brazil | Leopoldina | <1908 | BM | Brown 1976: 15c | F1? |
| 23 | H. ethilla narcaea | H. besckei | m | Brazil | Sta. Cat.: R. Grande do Sul |  | Wien | Holzinger & Holzinger 1994: 18,5b | F1 |
| 24 | H. ethilla narcaea | H. besckei | m | Brazil | Sta. Catarina: Joinville | 1985 | Neukirchen |  | F1 |
| 25 | H. ethilla narcaea | H. besckei | m | Brazil | Sta. Catarina: Joinville | 1981 | Para |  | F1 |
| 26 | H. ethilla narcaea | H. besckei | m | Brazil | Rio de Janeiro: Imbarie | 1963 | Para |  | F1 |
| 27 | H. ethilla narcaea | H. besckei | f | Brazil | Sta. Catarina: Agrolandia | <1997 | Para |  | F1 |
| 28 | H. ethilla narcaea | H. besckei | f | Brazil | SP: Horto Forestal | 1947 | Wien |  | BC->besckei |
| 29 | H. melpomene rosina | H. cydno galanthus | m | Costa Rica | Río Sarapiquí 600m | 1993 | MNCR | Posla-Fuentes 1993 | BC->cydno |
| 30 | H. melpomene rosina | H. cydno galanthus | f | Costa Rica | Río Sarapiquí | 1993 |  | Posla-Fuentes 1993 | BC->cydno |
| 31 | H. melpomene rosina | H. cydno galanthus | f | Costa Rica | Limón: Guácimo, San Luis | 1993 | BM |  | F1 |
| 32 | H. melpomene rosina | H. cydno chioneus | m | Panamá | Canal Zone: Pipeline Rd | 1979 | USNM |  | BC->cydno |
| 33 | H. melpomene cythera | H. cydno alithea | m | Ecuador | Balzapamba 800m | <1906 | BM | Ackery & Smiles 1976:184 | BC->melpomene |
| 34 | H. melpomene cythera | H. cydno alithea | m | Ecuador | Los Ríos: Río Palenque | 1973 | Allyn |  | F1 |
| 35 | H. melpomene cythera | H. cydno alithea | m | Ecuador | Pichincha: Alluriquín | 1989 | MHN, Lima |  | F1 |
| 36 | H. melpomene cythera | H. cydno alithea | m | Ecuador | Palmar 100m | ~1905 | BM |  | BC->melpomene |
| 37 | H. melpomene cythera | H. cydno alithea | m | Ecuador | Palmar 100m | ~1905 | BM |  | BC->melpomene |
| 38 | H. melpomene cythera | H. cydno alithea | m | Ecuador | Río Bamba | 1931 | BM |  | BC->melpomene |
| 39 | H. melpomene cythera | H. cydno alithea | m | Ecuador | Pichincha: S. Miguel | 1993 | Kapan |  | BC->melpomene |
| 40 | H. melpomene cythera | H. cydno alithea | m | Ecuador | Pichincha: El Padrino | 1994 | Kapan |  | BC->melpomene |
| 41 | H. melpomene {euryas} | H. cydno cydno | m | Colombia | Bogotá | <1906 | BM | Ackery & Smiles 1976:277 | BC->cydno |
| 42 | H. melpomene {euryas} | H. cydno cydno | m | Colombia | Caldas: Victoria | 1966 | Schmidt-M/IAvH | Salazar 1993:3 | BC->cydno |
| 43 | H. melpomene {euryas} | H. cydno cydno | m | Colombia | Muzo 400-800m | <1912 | BM | Ackery & Smiles 1976:278 | F1 |
| 44 | H. melpomene {euryas} | H. cydno cydno | m | Colombia | Boyacá: Otanche | 1986 | Neukirchen |  | F1 |
| 45 | H. melpomene {euryas} | H. cydno cydno | m | Colombia |  | <1892 | BM | Ackery & Smiles 1976:279 | F1 |
| 46 | H. melpomene {euryas} | H. cydno cydno | m | Colombia | Santa Fé de Bogotá | 1907 | BM |  | F1 |
| 47 | H. melpomene {euryas} | H. cydno cydno | m | Colombia |  | <1906 | BM |  | F1 |
| 48 | H. melpomene {euryas} | H. cydno cydno | m | Colombia | Muzo 400-800m | <1912 | BM |  | F1 |
| 49 | H. melpomene {euryas} | H. cydno cydno | m | Colombia | Sta. Fé de Bogotá | <1930 | BM |  | F1 |
| 50 | H. melpomene {euryas} | H. cydno cydno | m | Colombia | Boyacá: Otanche | 1980 | Schmidt-M/IAvH | Salazar 1993:4 | F1 |
| 51 | H. melpomene {euryas} | H. cydno cydno | m | Colombia | Caldas: Victoria | 1960 | Schmidt-M/IAvH |  | F1 |
| 52 | H. melpomene {euryas} | H. cydno cydno | m | Colombia | Caldas: 1.8 km N. Victoria | 1969 | Stuttgart |  | F1 |
| 53 | H. melpomene {euryas} | H. cydno cydno | m | Colombia | Boyacá: Otanche | 1990 | Schmidt-M/IAvH | Salazar 1993:5 | F1 |
| 54 | H. melpomene {euryas} | H. cydno cydno | m | Colombia | Boyacá: Otanche 500m | 1986 | Neukirchen |  | F1 |
| 55 | H. melpomene {euryas} | H. cydno cydno | m | Colombia | Cund.: Verag. |  | Wien |  | F1 |
| 56 | H. melpomene {euryas} | H. cydno cydno | f | Colombia | Boyacá: Otanche | 2001 | Le Crom |  | BC->cydno |
| 57 | H. melpomene {euryas} | H. cydno cydno | m | Colombia | Boyacá: Otanche | 1999 | Le Crom |  | BC->cydno |
| 58 | H. melpomene {euryas} | H. cydno cydno | m | Colombia | Boyacá: Otanche | 1985 | Le Crom |  | F1 |
| 59 | H. melpomene {euryas} | H. cydno cydno | m | Colombia | Boyacá: Otanche | 1986 | Le Crom |  | F1 |
| 60 | H. melpomene {euryas} | H. cydno cydno | m | Colombia | Boyacá: Otanche | 1988 | Le Crom |  | F1 |
| 61 | H. melpomene {euryas} | H. cydno cydno | f | Colombia | Boyacá: Otanche | 1983 | Le Crom |  | F1? |
| 62 | H. melpomene {euryas} | H. cydno cydno | m | Colombia | Boyacá: Otanche | 1993 | Le Crom |  | F1 |
| 63 | H. melpomene {euryas} | H. cydno cydno | m | Colombia | Boyacá: Otanche | 1991 | Le Crom |  | F1 |
| 64 | H. melpomene {euryas} | H. cydno cydno | m | Colombia | Huila: S. José Isnos | 1990 | Wien |  | BC->cydno |
| 65 | H. melpomene {euryas} | H. cydno hermogenes | f | Colombia | N. Granade | <1925 | BM | Ackery & Smiles 1976:268 | F1 |
| 66 | H. melpomene vulcanus | H. cydno ?chioneus | m | Colombia | (W. Colombia) | <1928 | ?Wien | Holzinger & Holzinger 1994: 35,4b | BC->cydno |
| 67 | H. melpomene vulcanus | H. cydno chioneus | m | Colombia | (W. Colombia) |  | Krüger | [Tomas Pyrcz photo] | BC->cydno |
| 68 | H. melpomene vulcanus | H. cydno zelinde | m | Colombia | Chocó: Río San Juan | 1988 | Neukirchen |  | BC->cydno |
| 69 | H. melpomene vulcanus | H. cydno zelinde | m | Colombia | Chocó: Río San Juan | 1988 | Neukirchen |  | F1 |
| 70 | H. melpomene vulcanus | H. cydno zelinde | m | Colombia | Chocó: Río San Juan | 1988 | Neukirchen |  | F1 |
| 71 | H. melpomene vulcanus | H. cydno zelinde | f | Colombia | Chocó: Río San Juan | 1988 | Neukirchen |  | F1 |
| 72 | H. melpomene vulcanus | H. cydno zelinde | m | Colombia | Valle: Cali | 1973 | Mast de Maeght |  | F1 |
| 73 | H. melpomene vulcanus | H. cydno zelinde | f | Colombia | Valle: Isla Punta Soldado | 2000 | Efraín Henao |  | F1 |
| 74 | H. melpomene vulcanus | H. cydno zelinde | f | Colombia | Valle: Río Calima | 1979 | A. Jaramillo |  | BC->melpomene |
| 75 | H. melpomene vulcanus | H. cydno weymeri f. weymeri | m | Colombia | Valle: Cali | 1969 | Mast de Maeght |  | BC->cydno |
| 76 | H. melpomene vulcanus | H. cydno weymeri f. gustavi | m | Colombia | Valle: Río Dagua | 1987 | Neukirchen |  | BC->cydno |
| 77 | H. melpomene vulcanus | H. cydno weymeri f. gustavi | m | Colombia | Valle: Río Dagua |  | Neukirchen |  | BC->cydno |
| 78 | H. melpomene vulcanus | H. cydno weymeri f. weymeri | f | Colombia | Valle: Río Dagua | 1969 | Wien |  | BC->cydno |
| 79 | H. melpomene vulcanus | H. cydno weymeri f. weymeri | m | Colombia | Valle: Río Dagua | 1968 | Mast de Maeght |  | BC->cydno |
| 80 | H. melpomene vulcanus | H. cydno weymeri f. weymeri | m | Colombia | Valle: Río Dagua | 1967 | Mast de Maeght |  | BC->cydno |
| 81 | H. melpomene vulcanus | H. cydno zelinde | m | Colombia | Valle: Río Dagua | ~1970 | Mast de Maeght |  | F1 |
| 82 | H. melpomene vulcanus | H. cydno weymeri f. weymeri | m | Colombia | Valle: Río Dagua | 1968 | Mast de Maeght |  | BC->cydno |
| 83 | H. melpomene vulcanus | H. cydno weymeri f. weymeri | m | Colombia | Valle: Río Dagua | 1966 | Mast de Maeght |  | BC->cydno |
| 84 | H. melpomene vulcanus | H. cydno weymeri f. weymeri | m | Colombia | Valle: Río Dagua | 1966 | Mast de Maeght |  | BC->cydno |
| 85 | H. melpomene vulcanus | H. cydno zelinde | f | Colombia | Valle: Río Calima | 1988 | Neukirchen |  | BC->cydno |
| 86 | H. melpomene vulcanus | H. cydno zelinde | f | Colombia | Valle: Río Calima | 1988 | Neukirchen |  | BC->cydno |
| 87 | H. melpomene melpomene | H. cydno cordula | f | Venezuela | Táchira: PN Paramillo | 2002 | Linares |  | BC->cydno |
| 88 | H. melpomene melpomene | H. cydno cordula | f | Venezuela | Táchira: PN Paramillo | 2002 | Linares |  | BC->cydno |
| 89 | H. melpomene melpomene | H. cydno cordula | m | Venezuela | Táchira: PN Paramillo | 2002 | Linares |  | BC->cydno |
| 90 | H. melpomene melpomene | H. cydno cordula | f | Venezuela | Táchira: PN Paramillo | 2002 | Linares |  | BC->cydno |
| 91 | H. melpomene melpomene | H. cydno cordula | f | Venezuela | Táchira: PN Paramillo | 2002 | Linares |  | BC->cydno |
| 92 | H. melpomene melpomene | H. cydno cordula | f | Venezuela | Táchira: Río Negro | 1980 | Maracay | Brown & Fernandez 1984:176 | F1 |
| 93 | H. melpomene melpomene | H. cydno cordula | m | Venezuela | ?Táchira | ~1980 | Romero |  | BC->cydno |
| 94 | H. melpomene melpomene | H. cydno gadouae | m | Venezuela | ?Táchira | ~1980 | Romero |  | F1 |
| **95** | H. melpomene melpomene | H. cydno barinasensis | m | Venezuela | Barinitas, Barinas | <1984 | Mattei | Brown & Fernandez 1984:177 | F1 |
| **96** | H. melpomene melpomene | H. cydno barinasensis | f | Venezuela | ?Barinitas, Barinas |  | Mattei |  | BC->cydno |
| 97 | H. melpomene melpomene | H. heurippa | f | Colombia | Cund.: Chirijara | 1991 | Schmidt-M/IAvH | Salazar 1993:7 | BC->heurippa |
| 98 | H. cydno galanthus | H. pachinus | m | Costa Rica | Turrialba |  | Wien | Holzinger & Holzinger 1994:35,2b | BC->cydno |
| 99 | H. cydno galanthus | H. pachinus | m/f | Costa Rica | Río Sarapiquí | ~1980 | Gilbert | Gilbert 2001:5 | BC->cydno |
| 100 | H. cydno galanthus | H. pachinus | m | Costa Rica | Heredia: La Selva Biol Stn | 2002 | Kronforst |  | BC->cydno |
| 101 | H. himera | H. erato cyrbia | m | Ecuador | El Oro: Guayquichuma | 1982 | Descimon | Descimon &c 1984: 1.2 (#1) | F1 |
| 102 | H. himera | H. erato cyrbia | m | Ecuador | El Oro: Guayquichuma | 1982 | Descimon | Descimon &c 1984: (#2) | F1 |
| 103 | H. himera | H. erato cyrbia | m | Ecuador | El Oro: Guayquichuma | 1982 | Mast de Maeght | Descimon &c 1984: (#3) | F1 |
| 104 | H. himera | H. erato cyrbia | m | Ecuador | El Oro: Guayquichuma | 1982 | Descimon | Descimon &c 1984: 1.4 (#4) | BC->erato |
| 105 | H. himera | H. erato cyrbia | m | Ecuador | El Oro: Guayquichuma | 1982 | Descimon | Descimon &c 1984: 1.5 (#5) | BC->erato |
| 106 | H. himera | H. erato cyrbia | m | Ecuador | El Oro: Guayquichuma | 1982 | Descimon | Descimon &c 1984: 1.6 (#6) | BC->himera |
| 107 | H. himera | H. erato cyrbia | m | Ecuador | El Oro: Guayquichuma | 1993 | Mallet | Jiggins &c 1996: #7 | F1 |
| 108 | H. himera | H. erato cyrbia | m | Ecuador | El Oro: Guayquichuma | 1993 | Mallet | Jiggins &c 1996: #8 | F1 |
| 109 | H. himera | H. erato cyrbia | m | Ecuador | El Oro: Guayquichuma | 1993 | Mallet | Jiggins &c 1996: #9 | BC->erato |
| 110 | H. himera | H. erato cyrbia | m | Ecuador | El Oro: Guayquichuma | 1993 | Neukirchen | Jiggins &c 1996: 4B (#10) | BC->himera |
| 111 | H. himera | H. erato cyrbia | m | Ecuador | El Oro: Guayquichuma | 1993 | Neukirchen | Jiggins &c 1996: 4A (#11) | F1 |
| 112 | H. himera | H. erato cyrbia | m | Ecuador | El Oro: Guayquichuma | 1993 | Neukirchen | Jiggins &c 1996: 4F (#12) | BC->erato |
| 113 | H. himera | H. erato cyrbia | m | Ecuador | El Oro: Guayquichuma | 1993 | Neukirchen | Jiggins &c 1996: #13 | BC->erato |
| 114 | H. himera | H. erato cyrbia | f | Ecuador | El Oro: Guayquichuma | 1993 | Neukirchen | Jiggins &c 1996: 4E (#14) | BC->erato |
| 115 | H. himera | H. erato cyrbia | m | Ecuador | El Oro: Guayquichuma | 1993 | Neukirchen | Jiggins &c 1996: 4C (#15) | BC->himera |
| 116 | H. himera | H. erato cyrbia | m | Ecuador | El Oro: Guayquichuma | 1993 | Neukirchen | Jiggins &c 1996: #16 | F1 |
| 117 | H. himera | H. erato cyrbia | m | Ecuador | El Oro: Guayquichuma | 1993 | Neukirchen | Jiggins &c 1996: 4D (#17) | BC->erato |
| 118 | H. himera | H. erato cyrbia | f | Ecuador | El Oro: Guayquichuma | 1993 | Neukirchen | Jiggins &c 1996: #18 | BC->erato |
| 119 | H. himera | H. erato cyrbia | m | Ecuador | El Oro: Guayquichuma | 1994 | Jiggins &c | Jiggins &c 1996: #19 | F1 |
| 120 | H. himera | H. erato cyrbia | m | Ecuador | El Oro: Guayquichuma | 1994 | Jiggins &c | Jiggins &c 1996: #20 | F1 |
| 121 | H. himera | H. erato cyrbia | m | Ecuador | El Oro: Guayquichuma | 1994 | Jiggins &c | Jiggins &c 1996: #21 | F1 |
| 122 | H. himera | H. erato cyrbia | m | Ecuador | El Oro: Guayquichuma | 1994 | Jiggins &c | Jiggins &c 1996: #22 | F1 |
| 123 | H. himera | H. erato cyrbia | m | Ecuador | El Oro: Guayquichuma | 1994 | Jiggins &c | Jiggins &c 1996: #23 | BC->himera |
| 124 | H. himera | H. erato cyrbia | m | Ecuador | El Oro: Guayquichuma | 1994 | Jiggins &c | Jiggins &c 1996: #24 | F1 |
| 125 | H. himera | H. erato cyrbia | m | Ecuador | El Oro: Guayquichuma | 1994 | Jiggins &c | Jiggins &c 1996: 4H (#25) | BC->himera |
| 126 | H. himera | H. erato cyrbia | m | Ecuador | El Oro: Guayquichuma | 1994 | Jiggins &c | Jiggins &c 1996: #26 | BC->erato |
| 127 | H. himera | H. erato cyrbia | f | Ecuador | El Oro: Guayquichuma | 1994 | Jiggins &c | Jiggins &c 1996: #27 | BC->erato |
| 128 | H. himera | H. erato cyrbia | f | Ecuador | El Oro: Guayquichuma | 1994 | Jiggins &c | Jiggins &c 1996: #28 | BC->erato |
| 129 | H. himera | H. erato cyrbia | m | Ecuador | El Oro: Guayquichuma | 1994 | Jiggins &c | Jiggins &c 1996: #29 | BC->erato |
| 130 | H. himera | H. erato cyrbia | m | Ecuador | El Oro: Guayquichuma | 1994 | Jiggins &c | Jiggins &c 1996: #30 | BC->erato |
| 131 | H. himera | H. erato cyrbia | m | Ecuador | El Oro: Guayquichuma | 1994 | Jiggins &c | Jiggins &c 1996: #31 | BC->himera |
| 132 | H. himera | H. erato cyrbia | m | Ecuador | El Oro: Guayquichuma | 1994 | Jiggins &c | Jiggins &c 1996: #32 | F1 |
| 133 | H. himera | H. erato cyrbia | m | Ecuador | El Oro: Guayquichuma | 1994 | Jiggins &c | Jiggins &c 1996: #33 | F1 |
| 134 | H. himera | H. erato cyrbia | m | Ecuador | El Oro: Guayquichuma | 1994 | Jiggins &c | Jiggins &c 1996: #34 | F1 |
| 135 | H. himera | H. erato cyrbia | m | Ecuador | El Oro: Guayquichuma | 1994 | Jiggins &c | Jiggins &c 1996: #35 | BC->erato |
| 136 | H. himera | H. erato cyrbia | m | Ecuador | El Oro: Guayquichuma | 1994 | Jiggins &c | Jiggins &c 1996: #36 | F1 |
| 137 | H. himera | H. erato cyrbia | f | Ecuador | El Oro: Chaguarpamba | 1994 | Jiggins &c | Jiggins &c 1996: #37 | F1 |
| 138 | H. himera | H. erato cyrbia | m | Ecuador | El Oro: Chaguarpamba | 1994 | Jiggins &c | Jiggins &c 1996: #38 | BC->erato |
| 139 | H. himera | H. erato cyrbia | f | Ecuador | El Oro: Guayquichuma | 1994 | Jiggins &c | Jiggins &c 1996: #39 | BC->himera |
| 1404 | H. himera | H. erato cyrbia | m | Ecuador | El Oro: Guayquichuma | 1994 | Jiggins &c | Jiggins &c 1996: #40 | F1 |
| 141 | H. himera | H. erato cyrbia | m | Ecuador | El Oro: Guayquichuma | 1994 | Jiggins &c | Jiggins &c 1996: 4G (#41) | BC->himera |
| 142 | H. himera | H. erato cyrbia | f | Ecuador | El Oro: Guayquichuma | 1994 | Jiggins &c | Jiggins &c 1996: #42 | BC->himera |
| 143 | H. himera | H. erato cyrbia | m | Ecuador | El Oro: Guayquichuma | 1994 | Jiggins &c | Jiggins &c 1996: #43 | BC->erato |
| 144 | H. himera | H. erato cyrbia | f | Ecuador | El Oro: Guayquichuma | 1996 | Jiggins &c | Mallet &c 1998: Tab1 | F1 |
| 145 | H. himera | H. erato cyrbia | m | Ecuador | El Oro: Guayquichuma | 1996 | Jiggins &c | Mallet &c 1998: Tab1 | BC->himera |
| 146 | H. himera | H. erato cyrbia | f | Ecuador | El Oro: Q. Zarza | 1996 | Jiggins &c | Mallet &c 1998: Tab1 | BC->himera |
| 147 | H. himera | H. erato cyrbia | m | Ecuador | El Oro: Guayquichuma | 1996 | Jiggins &c | Mallet &c 1998: Tab1 | F1 |
| 148 | H. himera | H. erato cyrbia | f | Ecuador | El Oro: Guayquichuma | 1996 | Jiggins &c | Mallet &c 1998: Tab1 | F1 |
| 149 | H. himera | H. erato cyrbia | m | Ecuador | El Oro: Guayquichuma | 1996 | Jiggins &c | Mallet &c 1998: Tab1 | F1 |
| 150 | H. himera | H. erato cyrbia | m | Ecuador | El Oro: Guayquichuma | 2001 | Boyer |  | F1 |
| 151 | H. himera | H. erato cyrbia | m | Ecuador | El Oro: Guayquichuma | 2001 | Boyer |  | BC->himera |
| 152 | H. himera | H. erato cyrbia | m | Ecuador | El Oro: Guayquichuma | 2001 | Boyer |  | BC->himera |
| 153 | H. himera | H. erato favorinus | m | Peru | San Martín: Rodriguez | 1984 | König | König 1986 | F1 |
| 154 | H. himera | H. erato favorinus | m | Peru | San Martín: Rodriguez | 1994 | Neukirchen |  | BC->erato |
| 155 | H. himera | H. erato favorinus | m | Peru | San Martín: Rodriguez | 1986 | Mallet |  | BC->erato |
| 156 | H. himera | H. erato favorinus | m | Peru | San Martín: Rodriguez | ~1986 | König |  | BC->erato |
| 157 | H. himera | H. erato lativitta | m | Peru | Amazonas: Aramango | <1990 | König |  | F1 |
| 158 | H. erato petiveranus | H. charithonia vasquezae | m | Mexico | Oaxaca: Chiltepec | 1970 | UNAM | De la Maza 1991:III,11 | F1? |
| 159 | H. charithonia | H. peruvianus | m | Ecuador | Pichincha: Río Toachi | 1996 | Jiggins | Jiggins & Davies 1998 | BC->charithonia |
| 160 | H. hecalesia octavia | H. hortense | m | Mexico | Oaxaca: Valle Nacional | 1987 | Neukirchen |  | F1 |
| 161 | H. hecalesia formosus | H. clysonymus montanus | m | Costa Rica | Cartago: Turrialba | 1995 | MNCR |  | F1 |

  

REFERENCES

Ackery, P. R., and R. L. Smiles. 1976. An illustrated
list of the type-specimens of the Heliconiinae (Lepidoptera: Nymphalidae)
in the British Museum (Natural History). Bull. Brit. Mus. (Nat. Hist.),
Entomol. 32:171-214.

Brown, K. S. 1976. An illustrated key to the silvaniform
Heliconius (Lepidoptera: Nymphalidae) with descriptions of new subspecies.
Trans. Amer. Entomol. Soc. 102:373-484.

Brown, K. S., and F. Fernandez Yepez. 1985. Los Heliconiini
(Lepidoptera, Nymphalidae) de Venezuela. Bol. Entomol. Venez. N.S. 3:29-76.

de la Maza, R. 1991. Mariposas Mexicanas, 2nd ed.
Fondo de Cultura Económica, México, D.F.

Descimon, H., and J. Mast de Maeght. 1984. Semispecies
relationships between Heliconius erato cyrbia Godt. and H. himera Hew.
in southwestern Ecuador. J. Res. Lepid. 22:229-239.

Gilbert, L. E. 2003. Adaptive novelty through introgression
in Heliconius wing patterns: evidence for a shared genetic "tool box" from
synthetic hybrid zones, and a theory of diversification. Pp. 281-318 in
C. L. Boggs, W. B. Watt, and P. R. Ehrlich, eds. Butterflies: Ecology and Evolution
Taking Flight. Univ. Chicago Press, Chicago.

Holzinger, H., and R. Holzinger. 1994. Heliconius
and Related Genera.  Lepidoptera: Nymphalidae.  The Genera Eueides,
Neruda and Heliconius. Sciences Nat, Venette, France.

Jiggins, C. D., and N. Davies. 1998. Genetic evidence
for a sibling species of Heliconius charithonia (Lepidoptera: Nymphalidae).
Biol. J. Linn. Soc. 64:57-67.

Jiggins, C. D., W. O. McMillan, W. Neukirchen, and
J. Mallet. 1996. What can hybrid zones tell us about speciation? 
The case of Heliconius erato and H. himera (Lepidoptera: Nymphalidae).
Biol. J. Linn. Soc. 59:221-242. (hybrids #1-#43 above are recorded in Appendix)

König, F. 1986. Ein Heliconius erato himera
- Hybrid aus Nord-Peru (Lepidoptera, Heliconiinae). Zeitschr. Arbeitsgem.
Österr. Entomol. 38:49-50.

Mallet, J., W. O. McMillan, and C. D. Jiggins. 1998.
Estimating the mating behavior of a pair of hybridizing Heliconius species
in the wild. Evolution 52:503-510.

Posla-Fuentes, M. 1993. An unusual form of Heliconius
cydno from Costa Rica. Trop. Lepid. 4:92.

Salazar, J. A. 1993. Notes on some populations of
Heliconius heurippa in Colombia (Lepidoptera: Nymphalidae: Heliconiinae).
Trop. Lepid. 4:119-121.

Velez, J., and J. Salazar. 1991. Mariposas de Colombia.
Villegas Editoras, Bogotá, Colombia.
